# Structural and Functional Characterization of Mature Forms of Metalloprotease E495 from Arctic Sea-Ice Bacterium *Pseudoalteromonas* sp. SM495

**DOI:** 10.1371/journal.pone.0035442

**Published:** 2012-04-16

**Authors:** Hai-Lun He, Jun Guo, Xiu-Lan Chen, Bin-Bin Xie, Xi-Ying Zhang, Yong Yu, Bo Chen, Bai-Cheng Zhou, Yu-Zhong Zhang

**Affiliations:** 1 State Key Laboratory of Microbial Technology, Marine Biotechnology Research Center, Shandong University, Jinan, China; 2 SOA Key Laboratory for Polar Science, Polar Research Institute of China, Shanghai, China; Argonne National Laboratory, United States of America

## Abstract

E495 is the most abundant protease secreted by the Arctic sea-ice bacterium *Pseudoalteromonas* sp. SM495. As a thermolysin family metalloprotease, E495 was found to have multiple active forms in the culture of strain SM495. E495-M (containing only the catalytic domain) and E495-M-C1 (containing the catalytic domain and one PPC domain) were two stable mature forms, and E495-M-C1-C2 (containing the catalytic domain and two PPC domains) might be an intermediate. Compared to E495-M, E495-M-C1 had similar affinity and catalytic efficiency to oligopeptides, but higher affinity and catalytic efficiency to proteins. The PPC domains from E495 were expressed as GST-fused proteins. Both of the recombinant PPC domains were shown to have binding ability to proteins C-phycocyanin and casein, and domain PPC1 had higher affinity to C-phycocyanin than domain PPC2. These results indicated that the domain PPC1 in E495-M-C1 could be helpful in binding protein substrate, and therefore, improving the catalytic efficiency. Site-directed mutagenesis on the PPC domains showed that the conserved polar and aromatic residues, D26, D28, Y30, Y/W65, in the PPC domains played key roles in protein binding. Our study may shed light on the mechanism of organic nitrogen degradation in the Arctic sea ice.

## Introduction

Sea ices, an important part of polar oceans, critically influence the productivity of the polar oceans, global energy budgets, and atmosphere-ocean interactions in the Arctic and Antarctic zones [1]. Heterotrophic bacteria and unicellular algae represent the two major groups of sea-ice assemblages [2]. The heterotrophic bacteria in sea ices are responsible for degradation of organic biopolymers and redistribution of organic matter between components of the sea-ice ecosystem, which have ecological significance in carbon and nitrogen cycling.

*Pseudoalteromonas* sp. SM495 is a protease-secreting bacterium isolated from the Arctic sea ice. E495, the most abundant protease secreted by strain SM495, is a cold-adapted metalloprotease of the thermolysin family (M4) [3]. The M4 family is a large family of zinc metalloproteases in the MA(E) subclan of the MA clan. Most of the peptidases in the M4 family are bacterial extracellular metalloproteases [4]. The representative of the M4 family, thermolysin (EC 3.4.24.27) secreted by *Bacillus thermoproteolyticus*, has been studied in detail and extensively applied for the synthesis of the artificial sweetener aspartame [5]. Besides thermolysin, most of the M4 metalloproteases studied in detail are the virulence factors of some pathogens [6]–[8]. The M4 metalloproteases are extensively secreted by bacteria from various habitats including soil, sediment and sea ice etc. As extracellular proteases, the M4 metalloproteases have been proposed to play an important role in nitrogen cycling (e.g., degradation of environmental proteins) [9], [10]. However, compared to study on thermolysin and the metalloproteases which are the virulence factors of some pathogens, study on the characteristics and function of the M4 metalloproteases from other bacteria is rare, especially those from the bacteria derived from deep-sea and polar habitats.

E495 precursor comprises 730 amino acids. Besides the signal peptide and the propeptide which are cleaved off after maturation, E495 contains a catalytic domain and two C-terminal PPC domains in its precursor [3]. PPC domain is found in some members of metalloprotease families M4, M9 and M28, as well as serine protease family S8 [11]. The PPC domains are usually cleaved after secretion, and therefore, mature proteases usually contain no PPC domain. It has been suggested that the PPC domains may aid secretion/localization or inhibit the protease until needed [12], [13]. However, recently, the serine protease MCP-03 from *Pseudoalteromonas* sp. SM9913 was reported to have two PPC domains in its recombinant mature form [14].

Our previous study showed that the recombinant E495 containing only the catalytic domain has better cold-adaptation ability compared to vibriolysin MCP-02 from cold-adapted deep-sea bacterium *Ps.* sp. SM9913 and pseudolysin from mesophilic *Pseudomonas aeruginosa* PAO1 [3]. In this study, we found that wild E495 had three active forms in the culture of strain SM495. E495-M containing only the catalytic domain and E495-M-C1 containing the catalytic domain and one PPC domain were two stable mature forms, and E495-M-C1-C2 containing the catalytic domain and two PPC domains might be an intermediate. We purified the two mature forms of E495 from strain SM495 and studied their substrate specificity towards various proteins and synthetic peptides. Moreover, the C-terminal PPC domains of E495 was shown to have protein-binding ability, and the key residues in the PPC domains for protein binding were determined by site-directed mutation.

## Materials and Methods

### Experimental materials and strains

The sea ice core sample was taken from Canadian Basin, Arctic Ocean (sampling site: 75°28′52″N, 152°51′18″W) during the 2nd Chinese National Arctic Research Expedition, 2003 summer. The strain *Ps.* sp. SM495 was isolated from the sea ice core sample at the 122–124 cm depth from ice surface [3]. *Escherichia coli* DH5α and *E. coli* BL21(DE3) was purchased from Novagen (USA) and grown at 37°C on LB medium supplemented with ampicillin for the selection of transformants. C-phycocyanin (CPC) and allophycocyanin (APC) were extracted and purified from *Synechocystis* sp. strain PCC6803 with the methods described by Nies et al. [15] and Su et al. [16], respectively. Insoluble type I collagen fiber (bovine Achilles tendon) was purchased from Worthington Biochemical Co. (USA), alpha casein, azoalbumin, gamma globulin and elastin (bovine neck ligament) from Sigma (USA), gelatin from Boston Biomedical Inc (USA), skim milk from Becton Dickinson Company (USA) and synthetic substrates, FA-Gly-Phe-NH_2_ (FAGFA), FA-Gly-Leu-NH_2_ (FAGLA) and FA-Gly-Val-NH_2_ (FAGVA) from Bachem A (Bubendorf, Switzerland).

### Protease purification

Strain *Ps.* sp. SM495 was inoculated in the fermentation medium previously described [17] and cultivated in a 50/500 ml flask at 15°C, 200 rpm for 90 h. After cultivation, the culture was centrifuged at 10,000× g, 4°C for 20 min. The supernatant was precipitated with 60% ammonium sulfate. The precipitate was dissolved in 20 mM Tris-HCl (pH 8.5), and dialyzed against the same buffer. The dialyzed sample was loaded onto a DEAE-Sepharose Fast Flow column (Amersham Biosciences, USA) and the bound proteins were eluted with an increasing gradient NaCl (0–1.0 M). Fractions with high protease activity were collected and concentrated using ultrafiltration. Proteases E495-M and E495-M-C1 in the concentrated sample was further separated by Native-polyacrylamide gel electrophoresis (Native-PAGE). The purity of E495-M and E495-M-C1 was analyzed using 12.5% sodium dodecyl sulfate-polyacrylamide gel electrophoresis (SDS-PAGE). The recombinant proteases MCP-02 and pseudolysin were expressed and purified with the procedure previously described [3].

### Determination of N-terminal sequences and molecular weights

The proteases were transferred into 12.5% gel using SDS-PAGE and electroblotted onto polyvinylidene difluoride (PVDF) membrane (GE Healthcare, USA) as described by Matsudaira [18] using the Bio-Rad Transblot apparatus. Protein bound to the PVDF membrane was visualized by staining with Coomassie blue prior to excision of the bands for N-terminal sequence analysis. N-terminal sequence analysis was carried out by automated Edman degradation using a *Procise 491* protein sequencer (Applied Biosystems, USA). The molecular weights (MW) of the proteases were determined using an Ultraflex MALDI-TOF/TOF mass spectrometer (Bruker Daltonics, Germany).

### Electrophoresis and zymogram

SDS-PAGE was performed according to the method of Laemmli [19]. Native-PAGE was performed according to the method of Liu [20]. Zymogram was performed using a modified method of Chakrabarti [21]. In zymogram, gelatin (0.1%), as the protease substrate, was incorporated in SDS-polyacrylamide before the polymerization of acrylamide. After electrophoresis, the gel was rinsed in 0.1 M Tris-HC1 containing 2.5% Triton X-100 (pH 7.5) for 45 min to remove the SDS and regenerate protease activity. The gel was subsequently soaked in pre-warmed 50 mM Tris–HCl (pH 7.5) at 37°C for 3 h to allow the protease to digest gelatin. The gel was then stained with Coomassie Brilliant Blue. The protease band(s) became clearly visible as unstained band(s) against a blue background.

### Protease production of *Ps.* sp. SM495 in different media

Protease production of *Ps.* sp. SM495 in five kinds of media with different nitrogen sources was analyzed. Flasks were prepared with the fermentation medium previously described [17] and other four media: basic medium (0.2% yeast extract and artificial seawater, pH 8.0) with 0.3% (w/w) casein, 0.3% (w/w) gelatin, 0.3% (w/w) elastin powder or 0.3% (w/w) skim milk powder. Strain *Ps.* sp. SM495 was inoculated in the media and cultivated in a 50/500 ml flask at 15°C, 200 rpm for 90 h. After cultivation, the culture was centrifuged at 10,000× g, 4°C for 20 min. Fifteen microliters of each culture supernatant were subjected to zymogram to analyze the relative content of proteases in each culture.

### Analysis of E495 stability

To analyze the stability of E495 in the culture, the supernatant of the fermentation solution was incubated at 15°C for 15 min, 1 h, 3 h, 5 h, 10 h or 20 h. After incubation, the proteases in the solutions were analyzed with zymography. Zymography was performed as described above. To analyze the stability of the purified E495-M-C1 and E495-M, E495-M-C1 (0.05 mg/ml) and E495-M (0.04 mg/ml) were incubated at 4°C for 0, 5, 10, 20 d. After incubation, the samples were subjected to 12.5% SDS-PAGE to analyze the relative content of E495-M-C1 and E495-M in these samples.

### Protein determination and enzyme assays

The protein concentration was determined using the Bradford method with bovine serum albumin (Sigma, USA) as a standard. The protease activity to casein was determined with the method described previously [17]. The protease activity to azoalbumin was analyzed with Phillips' method [22]. The protease activities to dipeptides FAGFA, FAGLA and FAGVA were analyzed using Feder's method with a UV/VIS-550 spectrophotometer (Jasco, Japan) [23]. The collagenolytic and elastinolytic activities of proteases were determined using the method previously described [24]. The protease activity to gelatin was determined with the method provided by Worthington Biochemical Co. [25]. The degradation ability of E495-M and E495-M-C1 to CPC, APC and gamma globulin were analyzed using SDS-PAGE. These proteins (50 µg each) were digested with proteases E495-M and E495-M-C1 (0.3 µM each) at 37°C for 15 min (CPC), 30 min (APC) or 12 h (gamma globulin). Then, the digestion samples were analyzed using 12.5% SDS-PAGE.

### Expression and purification of the GST-fused PPC domains

The PPC domains (PPC1: E539-A609, PPC2: G647-G716 and PPC12: E539-G716) of E495 were amplified using PCR with previously constructed plasmid pET-22b-E495 as a template [3]. The PCR products were ligated with *Eco*RI/*Xho*I-linearized pGEX-4T-1 vectors (Pharmacia Biotech Inc., USA) to construct plasmids (pGEX-4T-1-PPC1, pGEX-4T-1-PPC2, pGEX-4T-1-PPC12) for expression of GST-fused PPC domains. All the expression plasmids were transformed into *E. coli* BL21(DE3) competent cells. Expression was induced with 0.2 mM Isopropyl-β-D-thiogalactopyranoside (IPTG) at 15°C for 16 h. The GST-fused PPC domains (GST-PPC1, GST-PPC2, GST-PPC12) were purified by glutathione-agarose chromatography (GE Healthcare, USA).

### Site-directed Mutagenesis of the PPC Domains

Site-directed mutagenesis was carried out by overlapping extension-PCR with the expression plasmids constructed above (pGEX-4T-1-PPC1, pGEX-4T-1-PPC2) as templates. Mutated sites (PPC1: F5, D26, D28, Y30, Y65; PPC2: F5, D26, D28, Y30, W65) were introduced by the primers with single mutation (Table S1). The mutated genes were subcloned into pGEX-4T-1 and transformed into *E. coli* BL21(DE3). After confirmed by enzyme digestion and nucleotide sequencing, all mutations were expressed and purified with the same condition as GST-fused PPC domains.

### Substrate binding assay by “pull-down” affinity head interaction

Substrate binding of GST-fused PPC domains was assayed using the “pull-down” affinity head interaction method described by Gary [26] with some modification. Two hundred microliter of 40% glutathione-agarose resin (GE Healthcare, USA) was mixed with 500 µl GST-fused PPC domain (or a mutant protein) and incubated at room temperature for 30 min to attach the fusion protein to the resin. The protein and resin complex was then washed ten times with wash buffer to remove the unbound protein completely. Five hundred microliter substrate (0.1 mg/ml CPC or casein) was added to the washed resin and incubated at 4°C with shaking for 12 h for binding of the GST-fused PPC domain to the substrate protein. After incubation, the mixtures were centrifuged at 3, 000× g, 4°C for 10 min. The amount of the substrate protein remaining in the supernatant was analyzed by 12.5% SDS-PAGE. The expressed GST protein instead of GST-fused PPC domain in the solution and the solution only containing the substrate protein and the resin were used as controls. Coomassie-stained gels were scanned using Epson Perfection V500 scanner (Seiko Epson, Japan) and the proteins in the gels were quantitated using ImageJ 1.43u software (NIH, USA).

### Substrate binding and kinetic constants assay by Surface Plasmon Resonance (SPR)

The interactions of PPC1, PPC2 and their corresponding mutations with CPC were analyzed at 25°C using a BIACORE 3000 instrument (GE Healthcare, USA). Biacore CM5 research grade sensor chips, Tween 20, and Biacore amine coupling kit were purchased from GE Healthcare (USA). CPC was immobilized on the CM5 chip using standard amine-coupling method [27] with Biacore amine-coupling kit (GE Healthcare, USA). And then PPC1, PPC2 or their corresponding mutations were injected on the captured receptor at a concentration of 2 µM with a flow rate of 20 µl/min. The chip was regenerated after each cycle using the running phosphate buffer (20 mM, with 0.005% Tween 20, pH 8.0). On the same chip, data were recorded on a control blank flow cell to account for nonspecific binding of the analyte to the matrix and for the change in refractive index. The obtained sensorgrams were evaluated using the BIA evaluation software package.

To analyze the kinetic constants, various concentrations of the PPC domains (0.25 µM, 0.5 µM, 1 µM, 2 µM, 4 µM, 8 µM) were passed over the chip surface at a flow rate of 30 µl/min. The surfaces were regenerated by injection of 20 mM NaOH at a flow rate of 30 µl/min prior to the next injection cycle. Kinetic constants (*k*_a_, *k*_d_ and *K*_D_) from the raw data were calculated by non-linear regression or equilibrium binding analysis using the BIAevaluation software version 4.1 (GE Healthcare, USA) supplied with the instrument.

**Figure 1:**
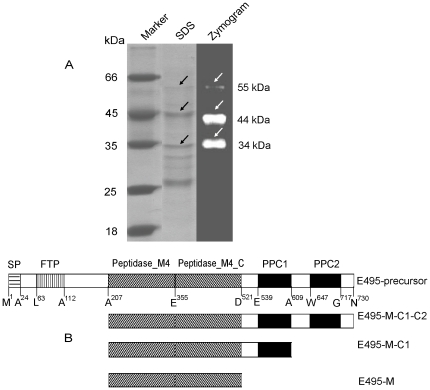
Analysis of multiple forms of protease E495 in strain SM495 culture. (A) The three forms of protease E495 in strain SM495 culture analyzed by SDS-PAGE and zymography. The bands of the three forms of E495 in the gels of SDS-PAGE and zymography are indicated by arrows. (B) The schematic diagram of domain architecture of E495 precursor and its three mature forms. The domain architecture of E495 precursor was analyzed with the CD-search service [28] available at NCBI (http://www.ncbi.nlm.nih.gov/Structure/cdd/cdd.shtml). The N- and C-terminal residues of each domain are shown. Abbreviations: SP, signal peptide; FTP, fungalysin/thermolysin propeptide motif; peptidase_M4, peptidase M4 catalytic domain; peptidase_M4_C, peptidase M4 alpha-helical domain; PPC1/PPC2: pre-peptidase C-terminal domain. The catalytic domain is composed of peptidase_M4 and peptidase_M4_C.

### Structure analysis of the PPC domains and their mutants with Circular Dichroism (CD)

CD spectra of the purified GST-fused PPC domains and their mutants in the same concentration (0.1 mg/ml) in 20 mM boric buffer (pH 8.0) were measured on a Jasco J810 spectropolarimeter (Japan) with a bandwidth of 2 nm, a response time of 1 s, and a scan speed of 200 nm/min. Each spectrum was an average of three scans monitored between 198 and 250 nm. The path length of the cuvette was 0.1 cm. The raw CD data were converted into mean residue ellipticity (deg cm^2^ dmol^−1^) at the entire wavelength using the relation: [*θ*] _λ_ = *θ*_λ_M_0_/10/*c*.

## Results

### Multiple mature forms of protease E495 secreted by *Ps.* sp. SM495

Zymogram gel analysis of the proteases secreted by *Ps.* sp. SM495 revealed three main protease bands, which were corresponding to three bands on the SDS-PAGE gel (Fig. 1A). The smallest protease has been confirmed to be one mature enzyme of E495 by N-terminal sequence analysis and its molecular weight (MW) has been determined by MS to be 33.4 kDa in our previous study [3]. The two larger protease bands in the gel were subjected to N-terminal sequence analysis. The results showed that they both have the same N-terminal sequence (ADATGPGGNLKTGLY) as E495-M, indicating that they were the different forms of protease E495 with different MW. Based on SDS-PAGE result, the largest form of E495 has a MW of about 55 kDa (Fig. 1A). Based on MS result, the smaller form of E495 has a MW of 43.9 kDa (Fig. S1). The sequence of E495 has been deduced from its gene sequence, and the domain architecture of E495 precursor has been predicted with the CD-search service available at NCBI [28]. As shown in Fig. 1B, E495 precursor contains a signal peptide (predicted with SignalP 3.0, http://www.cbs.dtu.dk/services/SignalP-3.0/), a propeptide, a catalytic domain and two PPC domains (PPC1, PPC2). It can be concluded from their N-terminal sequences that the three forms of E495 all contain the catalytic domain with or without the PPC domains. Since the largest form has a MW of 55 kDa (Fig. 1A) and the first N-terminal residue A207, according to the sequence and domain architecture of E495 precursor, the largest form has 524 amino acid residues (A207 to N730), containing the catalytic domain and the two PPC domains, which was named E495-M-C1-C2. Similarly, according to the MWof the smaller form (43.9 kDa, Fig. S1) and its N-terminal residue A207, it can be deduced form the sequence and domain architecture of E495 precursor that the smaller form has 403 amino acid residues (A207–A609), containing the catalytic domain and the PPC1 domain, which was named E495-M-C1. And according to the MWof the smallest form (33.4 kDa) and its N-terminal residue A207 analyzed in our previous study [3], the smallest form has 315 amino acid residues (A207-D521), only containing the catalytic domain, which was named E495-M. The domain architectures of E495-M-C1-C2, E495-M-C1 and E495-M are shown in Fig. 1B.

**Figure 2:**
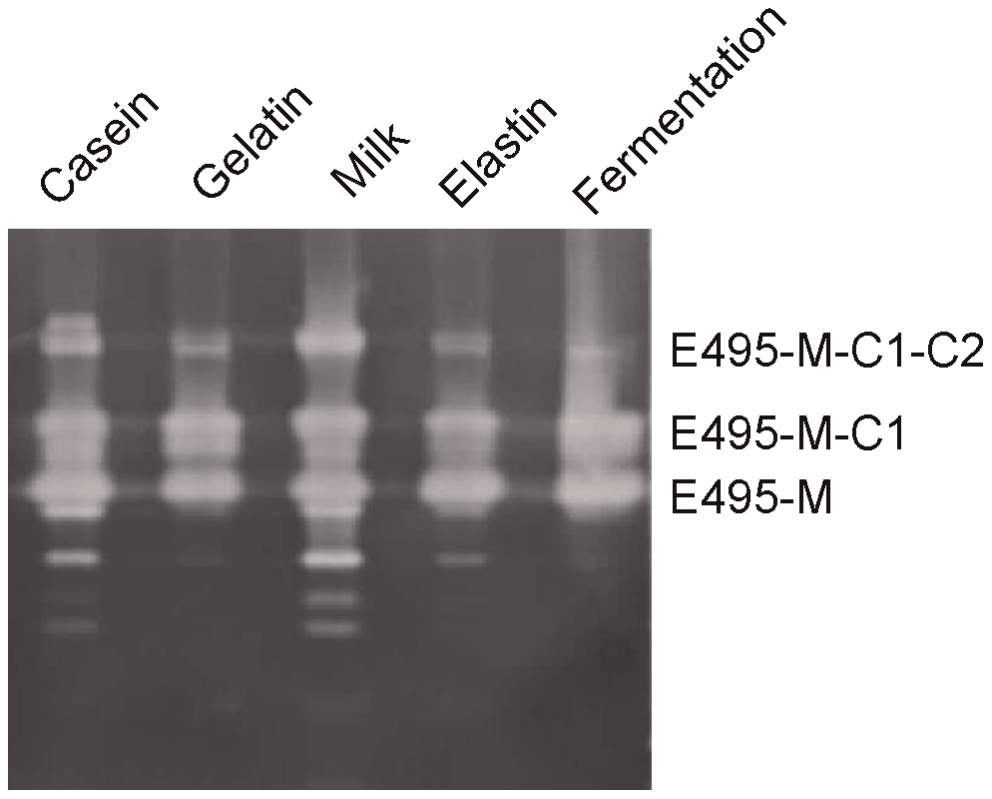
Protease production of *Ps.* sp. SM495 in different media. Strain SM495 was cultured at 15°C for 90 h in five different media as described in Materials and methods. Each culture supernatant (15 µl) was applied to zymogram to analyze the protease production.

**Figure 3:**
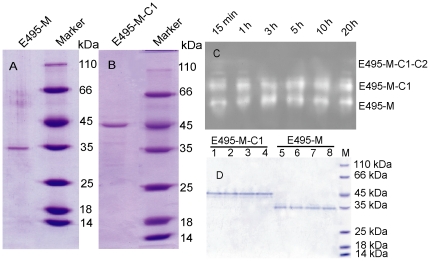
Purification and stability analysis of the mature forms of E495. (A) Purified E495-M. (B) Purified E495-M-C1. (C) The stability of the three forms of E495 in the fermentation medium at 15°C. The fermentation culture supernatants (15 µl) were incubated at 15°C for 15 min, 1 h, 3 h, 5 h, 10 h or 20 h, and then subjected to zymogram. (D) The stability of purified E495-M-C1 and E495-M at 4°C. The enzyme samples (0.05 mg/ml for E495-M-C1 and 0.04 mg/ml for E495-M) were incubated at 4°C for 0 (lane 1, lane 5), 5 (lane 2, lane 6), 10 (lane 3, lane 7) or 20 d (lane 4, lane 8), and then were subjected to SDS-PAGE analysis.

It can be seen from Fig. 1A that E495 was the most abundant extracellular protease secreted by *Ps.* sp. SM495 in the fermentation medium. To further investigate whether E495 is always the most abundant extracellular protease of *Ps.* sp. SM495 in different conditions, *Ps.* sp. SM495 was cultured in another 4 media with different nitrogen sources, and protease production of strain SM495 under these conditions was analyzed. Similar to the case in the fermentation medium, E495 was the most abundant extracellular protease of *Ps.* sp. SM495 in all other 4 media (Fig. 2). This result implies that E495 may be the most abundant extracellular protease of *Ps.* sp. SM495 in the natural sea ice environment.

### Purification and stability analysis of the three forms of protease E495

Efforts were made to purify all the three forms of E495 from the culture of strain SM495. E495-M-C1 and E495-M were purified (Fig. 3A and B), whereas the purified E495-M-C1-C2 could not be obtained because it was unstable in the culture according to the following stability analysis.

Zymogram analysis showed that the three forms of E495 were all active (Fig. 1A), indicating that they are not precursors. Since protease intermediates are usually quite unstable, the stability of E495-M-C1-C2 and E495-M-C1 was analyzed to determine whether they are intermediates or mature forms. As shown in Fig. 3C, the largest form E495-M-C1-C2 had low stability in fermentation culture at 15°C, suggesting that E495-M-C1-C2 was unstable and the PPC2 domain was easy to be cleaved off during fermentation culture. However, E495-M-C1 was as stable as E495-M in the fermentation culture at 15°C (Fig. 3C), and the purified E495-M-C1 was also as stable as the purified E495-M after incubation at 4°C for 20 d (Fig. 3D), indicating that the PPC1 domain was not cut off from E495-M-C1. Taken together, our results indicated that E495-M-C1-C2 might be an intermediate of E495, and both E495-M-C1 and E495-M were stable mature forms of E495.

### Substrate specificity of E495

The substrate specificity of E495-M towards several proteins and synthetic peptides was studied, and compared with that of MCP-02 and pseudolysin, two analogous metalloproteases of the M4 family. Synthetic peptides FAGFA, FAGLA and FAGVA are common substrates of the M4 metalloproteases. The three tested M4 metalloproteases had similar specificity to these peptides, with the highest catalytic efficiency (*k*_cat_/*K*_m_) towards FAGFA and the lowest towards FAGVA (Table 1). E495-M, MCP-02 and pseudolysin all could hydrolyze casein, CPC, APC and azoalbumin efficiently (Table 1). Towards algal proteins CPC and APC, the relative catalytic efficiency of E495-M was much higher than MCP-02 and pseudolysin (Fig. S2 A and B), while pseudolysin degraded azoalbumin much faster than E495-M and MCP-02 (Table 1). E495-M and MCP-02 had no activity towards elastin, while pseudolysin, a well-known elastinase, had high elastinolytic activity (Table 1). All the three proteases had a little activity towards gelatin and gamma globulin, and no activity towards insoluble type I collagen (Fig. S2 C, Table 1).

**Figure 4:**
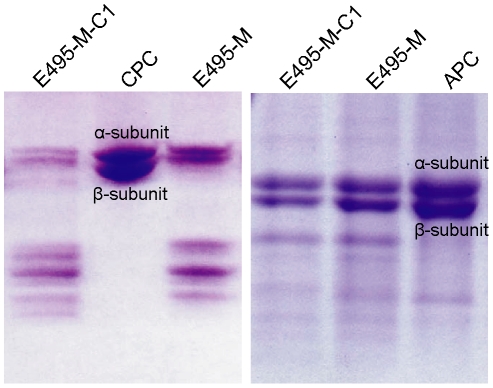
Comparison of the degradation ability of E495-M-C1 and E495-M to CPC (A) and APC (B). E495-M (0.3 µmol/L) and E495-M-C1 (0.3 µmol/L) were incubated at 37°C with 200 µg CPC for 15 min or 200 µg APC for 30 min, and then the hydrolysis products were analyzed by 12.5% SDS-PAGE. CPC and APC both contain two subunits α and β as shown in the figures.

**Table 1:** The substrate specificities of E495-M towards proteins and synthetic peptides.

Substrate	Catalytic efficiency or specific activity^a^		
	E495-M	MCP-02	Pseudolysin
CPC	+++	++	+
APC	+++	++	+
Gamma globulin	±	±	±
Casein (U/mg)	13800	10275	7061
Azoalbumin (U/mg)	94.75	110.3	244.5
Elastinorcein (U/mg)	0	0	91.1
Gelatin (U/mg)	1.06	1.13	4.99
Insoluble type I collagen fiber (U/mg)	0	0	0
FAGFA *k*_cat_/*K*_m_ (mM^−1^ s^−1^)	6.1	4.89	5.82
FAGLA *k*_cat_/*K*_m_ (mM^−1^ s^−1^)	4.35	1.78	0.86
FAGVA *k*_cat_/*K*_m_ (mM^−1^ s^−1^)	0.32	0.29	0.04

a : The activities of E495-M, MCP-02 and pseudolysin to casein, gelatin and azoalbumin were measured at 50°C, and to elastinorcein and insoluble type I collagen at 37°C with the methods described in Materials and Methods. The *k*_cat_/*K*_m_ values of E495-M, MCP-02 and pseudolysin to FAGFA, FAGLA and FAGVA were measured at 25°C with Feder's method [23]. The data represent the mean of three experimental repeats with SD≤5%. The hydrolysis of CPC, APC and gamma globulin by E495-M, MCP-02 and pseudolysin were performed at 37°C and analyzed by SDS-PAGE shown in Fig. S2. +++, the highest activity; ++, higher activity; +, low actitity, ±, a little activity.

### Comparison of the catalytic efficiency of E495-M and E495-M-C1 towards peptide FAGFA and proteins

In order to study the function of the PPC domains in E495, we compared the catalytic efficiency of E495-M and E495-M-C1 towards synthetic peptide FAGFA and proteins casein, CPC and APC. E495-M-C1 and E495-M showed similar catalytic efficiency towards the peptide FAGFA. However, E495-M-C1 showed higher catalytic efficiency towards proteins casein, CPC and APC than E495-M (Table 2, Fig. 4). In addition, compared to E495-M, E495-M-C1 showed similar *K*_m_ towards the peptide FAGFA, but lower *K*_m_ towards casein (Table 2), indicating that the C-terminal PPC domain in E495 could increase the affinity of the enzyme to protein substrate. These results suggested that the PPC domain may be helpful for the efficient binding of E495 to protein substrates, and therefore, improving the catalytic efficiency of the enzyme to proteins.

**Table 2:** Kinetic parameters of E495-M and E495-M-C1 towards peptide FAGFA and protein α-casein.

Substrate	*k*_cat_/*K*_m_ (mM^−1^ s^−1^)		*K*_m_ (mM)	
	E495-M	E495-M-C1	E495-M	E495-M-C1
FAGFA^a^	6.1	5.7	1.66	1.53
α-casein^b^	11.8	60.8	0.067	0.018

a The *K*_m_ and *k*_cat_/*K*_m_ values of E495-M and E495-M-C1 to FAGFA were determined at 25°C with Feder's method [23]. The data shown are the mean of three repeats with standard errors ≤5%.

b The *K*_m_ values of E495-M and E495-M-C1 to α-casein at 50°C were determined by non-linear fit analysis based on Michaelis–Menten equation shown in Fig. S3. *k*_cat_ values were calculated with the formula *k*_cat_ = *V*_max/_[*E*].

### Substrate-binding ability of the PPC domains of E495

**Figure 5:**
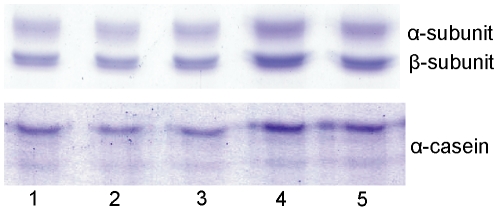
The binding ability of recombinant PPC1, PPC2 and PPC12 domains to CPC and α-casein. CPC contains two subunits α and β as shown in the figure. The binding ability of PPC domains to protein CPC and α-casein was analyzed using the “pull-down” affinity head interaction method described in Experimental Procedures. Lane 1, PPC1; lane 2, PPC2; lane 3, PPC12; lane 4, the control without any PPC domain; lane 5, the control with GST instead of PPC domain.

**Figure 6:**
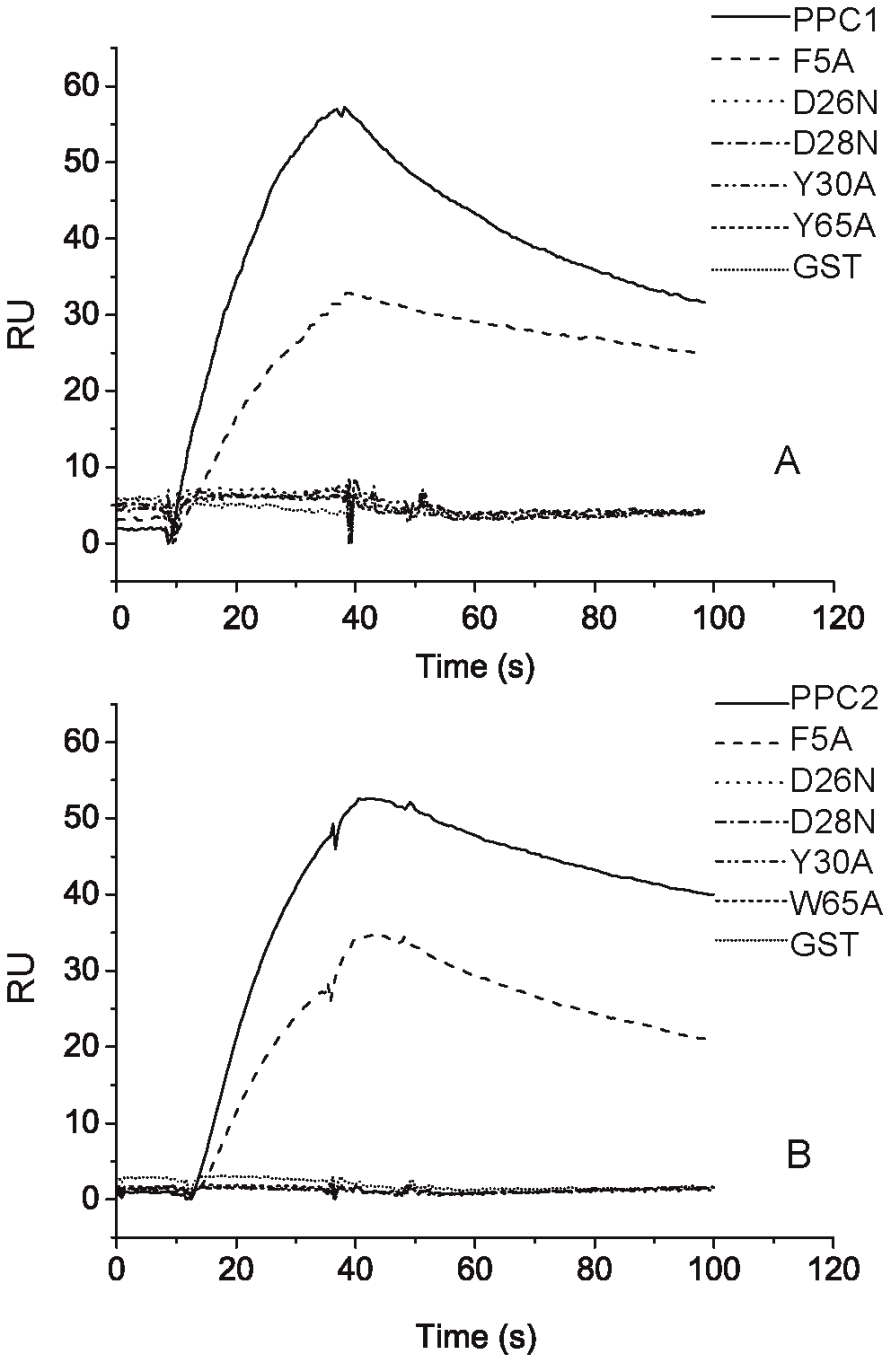
Analysis of the interaction between PPC domains and CPC by surface plasmon resonance (SPR). A, SPR spectra of PPC1 and its mutants. PPC1 or its mutants (2 µM) flowed over the surface of the CPC-coated chip with a rate of 20 µl/min at 25°C. B, SPR spectra of PPC2 and its mutants. PPC2 or its mutants (2 µM) flowed over the surface of the CPC-coated chip with a rate of 20 µl/min at 25°C.

**Figure 7:**
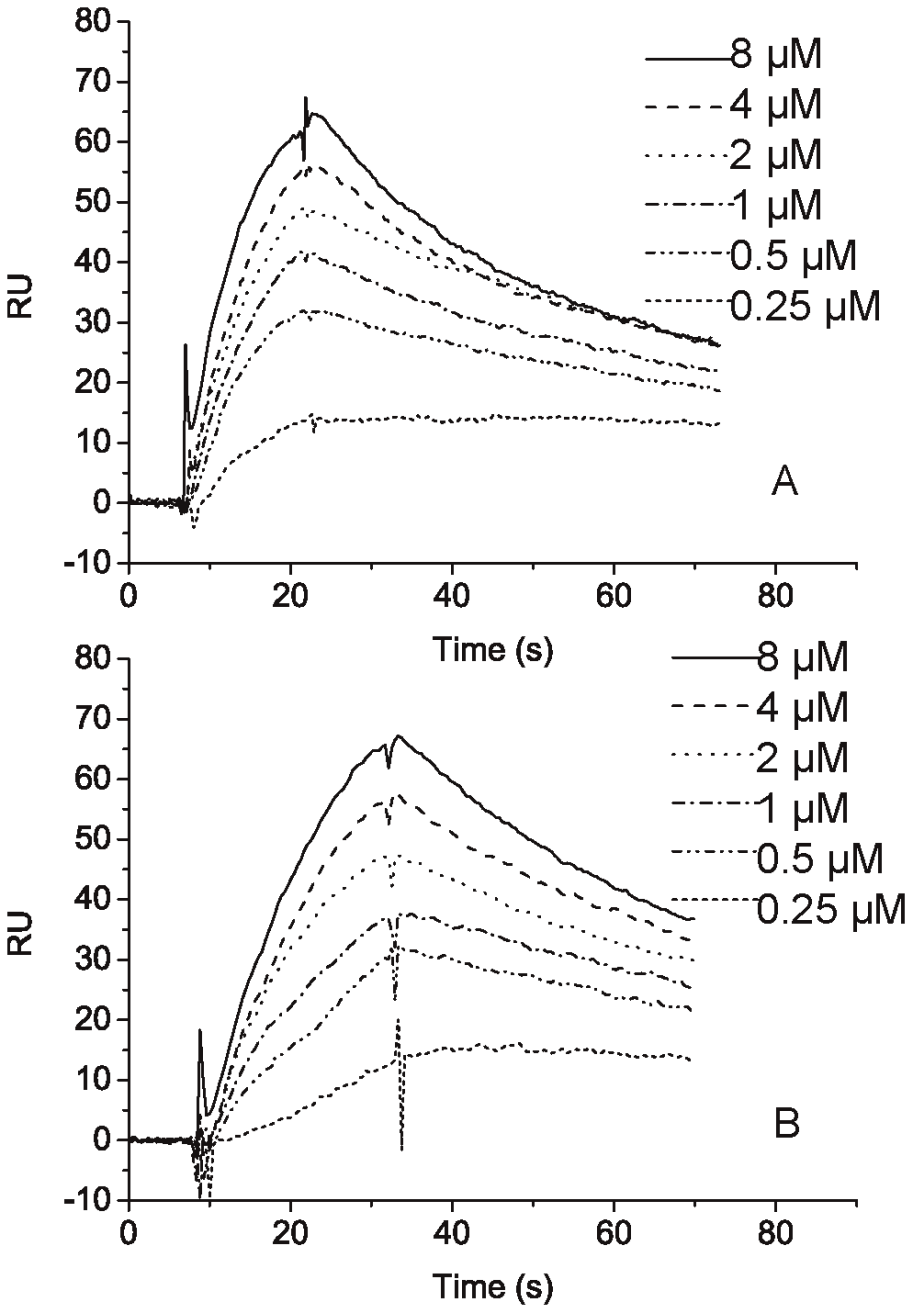
Analysis of kinetic constants by surface plasmon resonance (SPR). A, SPR spectra of PPC1 with different concentrations (0.25 µM, 0.5 µM, 1 µM, 2 µM, 4 µM, 8 µM). B, SPR spectra of PPC2 with different concentrations (0.25 µM, 0.5 µM, 1 µM, 2 µM, 4 µM, 8 µM). PPC1 or PPC2 (0.25 to 8 µM) flowed over the surface of the CPC-coated chip with a rate of 30 µl/min at 25°C.

In order to test whether the PPC domains of E495 has binding ability to protein substrate, the PPC domains of E495, PPC1, PPC2 and PPC12 (containing PPC1 and PPC2), were expressed as GST fusion proteins. The “pull-down” affinity head interaction assay was used to determine the binding ability of these fusion proteins to CPC and casein. As shown in Fig. 5, all the fused PPC domains, PPC1, PPC2 and PPC12, could bind to CPC and casein, whereas GST and glutathione-agarose resin had no binding ability to CPC and casein. Quantitive analysis showed that about 30% of CPC or casein was bound by the PPC domains (Table S2). Moreover, SPR analysis was performed to further analyze the protein substrate-binding ability of the PPC domains, which showed that both PPC1 and PPC2 domains had visible interaction with CPC, while GST did not have (Fig. 6). To quantitatively estimate the difference between PPC1 and PPC2 in their interaction with CPC, the profiles of GST-fused PPC binding to CPC were monitored as a function of reaction time on the basis of SPR signal (Fig. 7). Global fits of the data over a restricted range of analyte concentrations (0.25 to 8 µM) yielded estimates of kinetic constants. The *k*_a_, *k*_d_ and *K*_D_ values obtained by SPR analysis were summarized in Table 3. The overall kinetic data showed that both PPC domains were capable of binding to CPC. The *K*_D_ of PPC1 was lower than that of PPC2 (0.77 µM for PPC1, 2.47 µM for PPC2), indicating that PPC1 had higher affinity to CPC than PPC2. These results showed that the PPC domains in E495 have protein substrate-binding ability.

**Table 3:** Binding kinetic constants of recombinant PPC1 and PPC2 with CPC analyzed by SPR^a^.

PPC domain	*k*_a_ (1/Ms)	*k*_d_(1/s)	*K*_D_(µM)
PPC1	2.04×10^4^	1.58×10^−2^	0.77
PPC2	6.56×10^3^	1.62×10^−2^	2.47

a The kinetics of interaction between PPC and CPC was estimated by SPR (Fig. 7 A and B). *k*_a_, *k*_d_ and *K*_D_ were analyzed with a Biaevaluation software package (version 4.1, GE Healthcare, USA).

### Determination of the key residues in the PPC domains of E495 for protein binding

**Figure 8:**
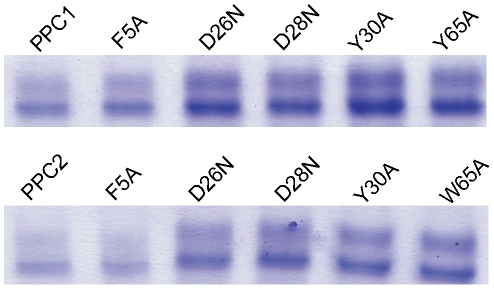
Effects of site-directed mutagenesis on the substrate-binding ability of PPC domains. A, PPC1 domain; B, PPC2 domain; CPC was as a substrate.

The sequences of the PPC domains in some bacterial metalloproteases were aligned to analyze the conserve amino acid residues (Fig. S4). Since the key residues responsible for protein binding are mostly reported to be polar and aromatic amino acids in many proteins [24], [29], [30], the conserved polar and aromatic residues, F5, D26, D28, Y30, Y65 in PPC1 and F5, D26, D28, Y30, W65 in PPC2, were selected for side-directed mutagenesis. The mutants, F5A, D26N, D28N, Y30A and Y/W65A were all expressed as GST fusion proteins, and their CPC-binding ability was analyzed by “pull-down” affinity head interaction and SPR. Results with similar trend were obtained from both experiments. While the mutation F5 to A only led to a little decrease in the CPC-binding ability of PPC domains, the other mutations apparently reduced the ability (Fig. 6, Fig. 8). To avoid that any mutation led to structure changes in the PPC domains to result in reduction in the substrate-binding ability, the structures of the PPC domains and their mutants were detected by CD. The results showed that no mutation caused notable structure change in the PPC domains (Fig. S5). Therefore, reduction in the substrate-binding ability of the mutants should result from amino acid replacement. These results suggested that the four conserved residues, D26, D28, Y30 and Y/W65, in the PPC1 and PPC2 domains of E495 play key roles in binding protein substrate.

## Discussion

The protease E495 secreted by the Arctic sea-ice bacterium *Ps.* sp. SM495 is a metalloprotease of the M4 family. E495 and some other proteases in this family, such as MCP-02 from *Ps.* sp. SM9913 and EmpI from *Ps.* sp. strain A28, have two C-terminal PPC domains in their precursors. Both MCP-02 and EmpI have one form of mature enzyme which contains only the catalytic domain [3], [31]. Although E495 has high identities (>90%) to these two M4 metalloproteases, we found that E495 has two mature forms, which have been rarely reported.

Although PPC domains are found in the precursors of many metalloproteases and serine proteases, there are very few mature proteases containing PPC domains [9], [32]. The function of the PPC domain in proteases is still largely unclear. The homologous C-terminal PPC domain of *Vibrio vulnificus* metalloprotease was reported to be essential for efficient attachment to insoluble protein substrates and erythrocyte membranes [33]. The C-terminal PPC domains of serine protease MCP-03 are unnecessary for enzyme secretion but have an inhibitory effect on MCP-03 catalytic efficiency and are essential for keeping MCP-03 thermostable [14]. The results in this study showed that the PPC domains from E495 had binding ability to protein substrates, which indicates that the C-terminal PPC domain in a protease can function as a binding domain to protein substrates. Moreover, although the two mature forms of E495, E495-M and E495-M-C1, had similar catalytic efficiency and affinity to oligopeptide substrate, E495-M-C1 had higher affinity and catalytic efficiency to proteins. This suggests that the C-terminal PPC domain in E495-M-C1 may be helpful in binding protein substrate, and therefore improving the catalytic activity of the enzyme towards proteins.

Many hydrolytic enzymes have a substrate binding domain (SBD), such as the cellulose-binding domain in cellulases [34], the collagen-binding domain in matrix metalloproteases [35], and the PKD domain in callagenolytic protease MCP-01 [36] and in chitinase A [37]. The key residues responsible for substrate binding in some of these SBD have been determined, which are all by means of side-directed mutation. It is shown that the key residues responsible for substrate binding in these SBD are usually conserved aromatic or polar amino acid residues [24], [34]. In this study, we used the same methods as those in these references to determine the key residues responsible for substrate binding in the PPC domains. Side-directed mutagenesis showed that the conserved polar and aromatic residues, D26, D28, Y30 and Y/W65, in the PPC domains play key roles in the binding of PPC domain to proteins. Tyrosine (Y), which contains huge side chain groups, helps to maintain the interaction by forming hydrogen bonds and hydrophobic force [38], [39]. Aspartate (D) might interact with the peptides ligand via making a direct salt bridge [40].

Because sea ice environment is difficult to simulate under laboratory conditions, many researches about sea ice bacteria secreting enzymes are cultured using synthesis or fermentation media under laboratory conditions [41]. In this study, we confirmed that the extracellular protease E495 was always the most abundant extracellular protease of *Ps.* sp. SM495 even in five different culture media, which may imply that E495 is the most abundant extracellular protease of *Ps.* sp. SM495 in the natural sea ice environment. The bacterial extracellular proteases in the Arctic sea ice ecosystem are mainly responsible for organic nitrogen degradation, whose mechanism is still unclear. Our results show that E495 has evolved high specific activity to many kinds of proteins, especially algal proteins, to be adapted to the sea ice environment. Moreover, E495 has more than one mature forms for protein degradation, which may be a strategy of strain SM495 to efficiently degrade the peptides and proteins in the sea ice. Therefore, as the most abundant extracellular protease of *Ps.* sp. SM495, E495 would play an important role in sea-ice organic nitrogen degradation and bacterial nutrition. Our study may shed light on the mechanism of organic nitrogen degradation in the Arctic sea ice.

## Supporting Information

Figure S1 Molecular weight of E495-M-C1 determined by mass spectroscopy. The molecular weight of E495-M-C1 was determined by using a Ultraflex MALDI-TOF/TOF mass spectrometer (Bruker Daltonics, Germany). The result showed that the molecular weight of E495-M-C1 is 43914 Da.
(TIF)

Figure S2 Comparison of the relative catalytic efficiency of E495, MCP-02 and pseudolysin to proteins CPC (A), APC (B) and gamma globulin (C). Each protease (0.3 µmol/L) was incubated at 37°C with CPC for 15 min, APC for 30 min, and gamma globulin for 12 h, respectively. Then, the hydrolysis products were analyzed on 12.5% SDS-PAGE gel. CPC and APC both contain two subunits α and β as shown in the figures.
(TIF)

Figure S3 Non-linear fit curves for α-casein hydrolysis by E495-M and E495-M-C1. The initial reaction rates were determined with 0–0.6 mM α-casein at 50°C.
(TIF)

Figure S4 Alignment of the PPC domains of E495 with those of the metalloprotease from *Ps.* sp. A28, the putative metalloproteinase from *Ps. tunicata* D2, and the metalloprotease II from *Ps. piscicida*. Identical residues are indicated by asterisks. Sequences were aligned using Clustal ×1.83.
(TIF)

Figure S5 CD spectra of GST-fused PPC domains and their mutants. The spectra were measured on a Jasco J-810 spectropolarimeter (Jasco Japan) with the method described in “Materials and Methods.”
(TIF)

Table S1 Oligonucleotide primers used to amplify PPC domains and their mutants.
(DOC)

Table S2 Quantitive analysis of the amount of CPC or casein bound by the PPC domains in the assay of the “pull-down” affinity head interaction^a^.
(DOC)
