# Supplementary figures and images for: Structural and Functional Characterization of Mature Forms of Metalloprotease E495 from Arctic Sea-Ice Bacterium Pseudoalteromonas sp. SM495
Source: PLoS One. 2012 Apr 16;7(4):e35442. doi: 10.1371/journal.pone.0035442 (PMC3327674; doi:10.1371/journal.pone.0035442)

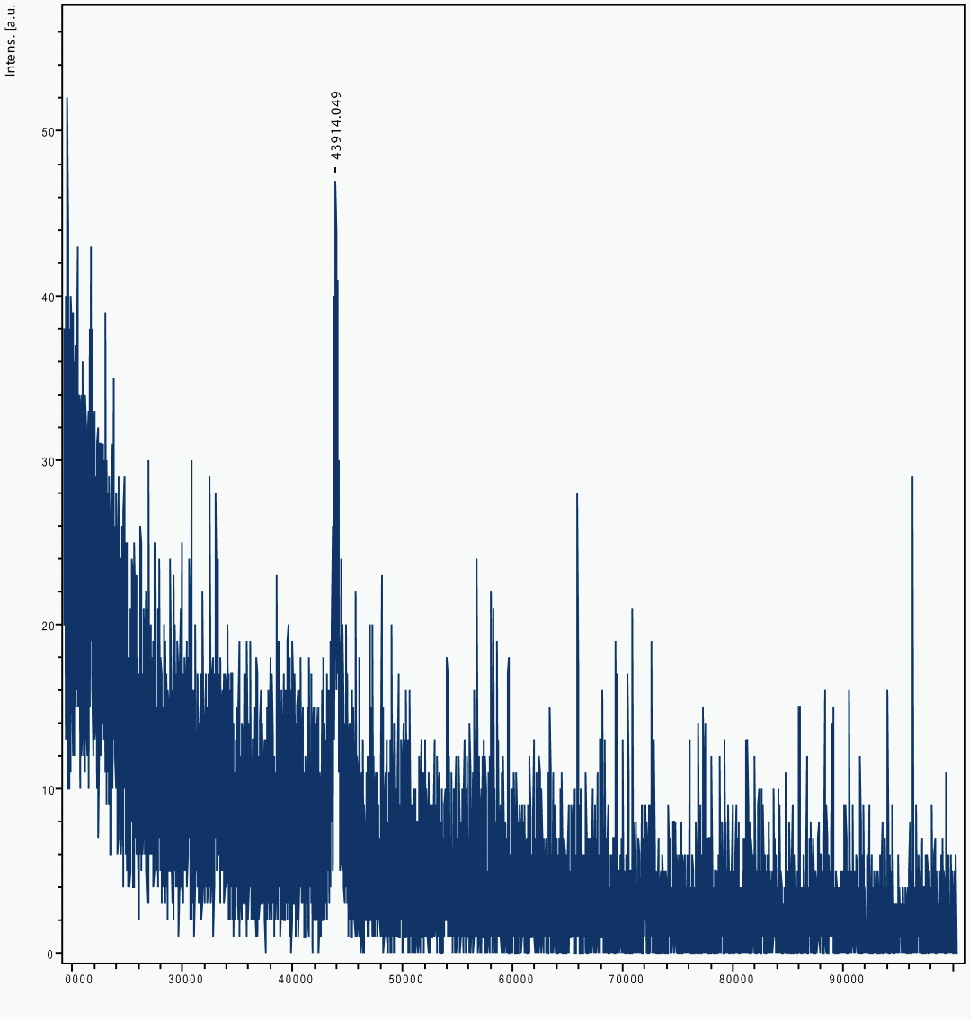

Supplement: Figure S1 — Molecular weight of E495-M-C1 determined by mass spectroscopy. The molecular weight of E495-M-C1 was determined by using a Ultraflex MALDI-TOF/TOF mass spectrometer (Bruker Daltonics, Germany). The result showed that the molecular weight of E495-M-C1 is 43914 Da. (TIF) [file pone.0035442.s001.tif]

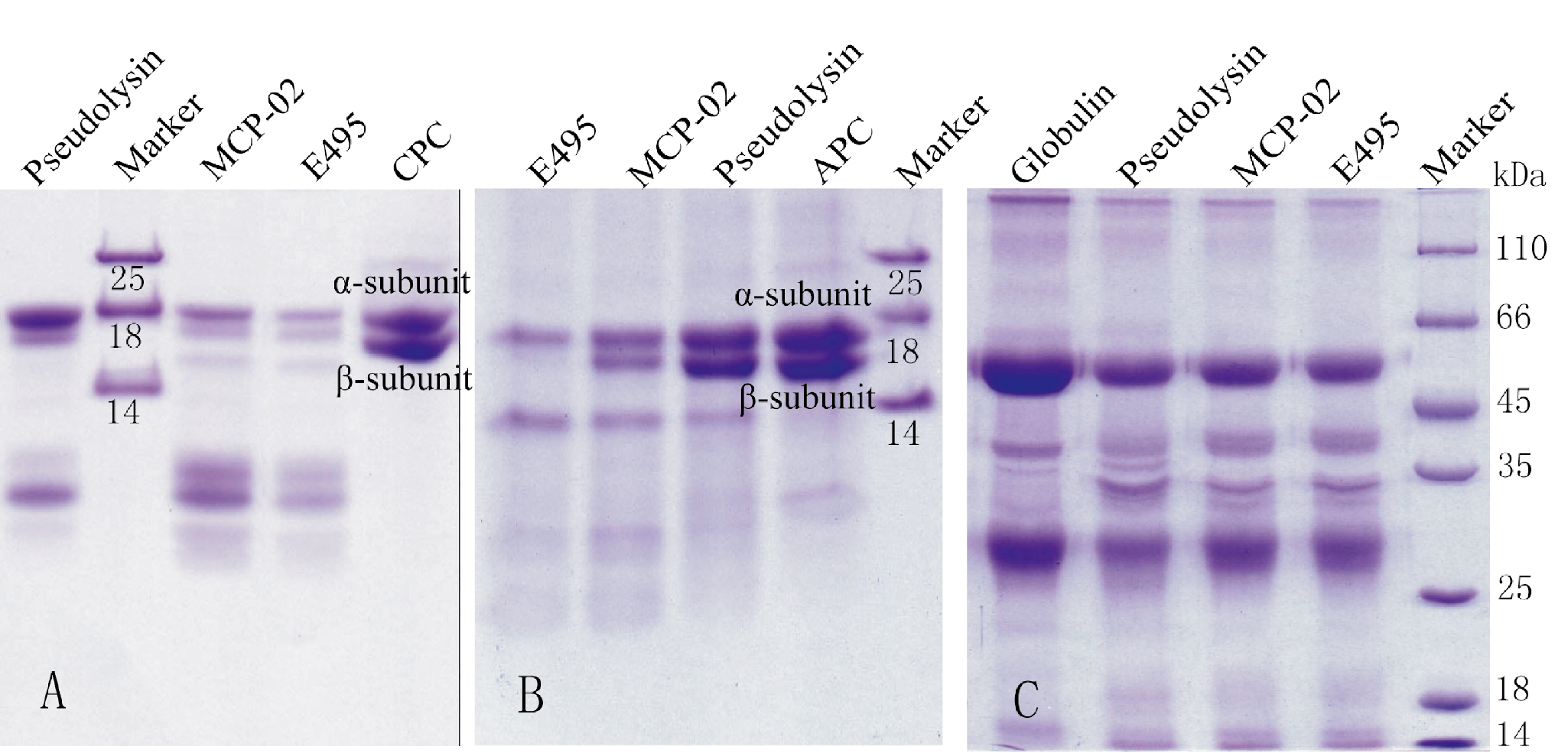

Supplement: Figure S2 — Comparison of the relative catalytic efficiency of E495, MCP-02 and pseudolysin to proteins CPC (A), APC (B) and gamma globulin (C). Each protease (0.3 µmol/L) was incubated at 37°C with CPC for 15 min, APC for 30 min, and gamma globulin for 12 h, respectively. Then, the hydrolysis products were analyzed on 12.5% SDS-PAGE gel. CPC and APC both contain two subunits α and β as shown in the figures. (TIF) [file pone.0035442.s002.tif]

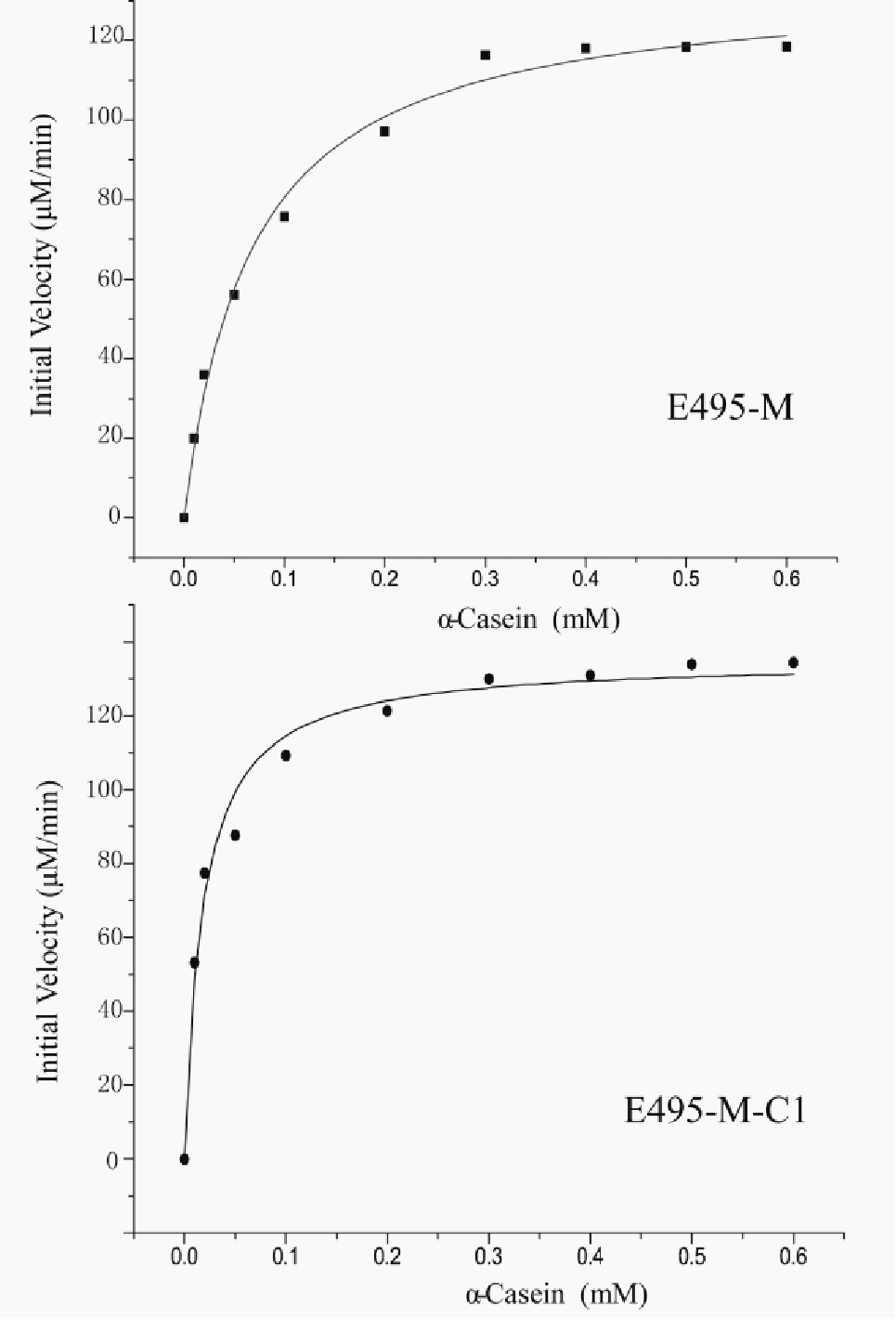

Supplement: Figure S3 — Non-linear fit curves for α-casein hydrolysis by E495-M and E495-M-C1. The initial reaction rates were determined with 0–0.6 mM α-casein at 50°C. (TIF) [file pone.0035442.s003.tif]

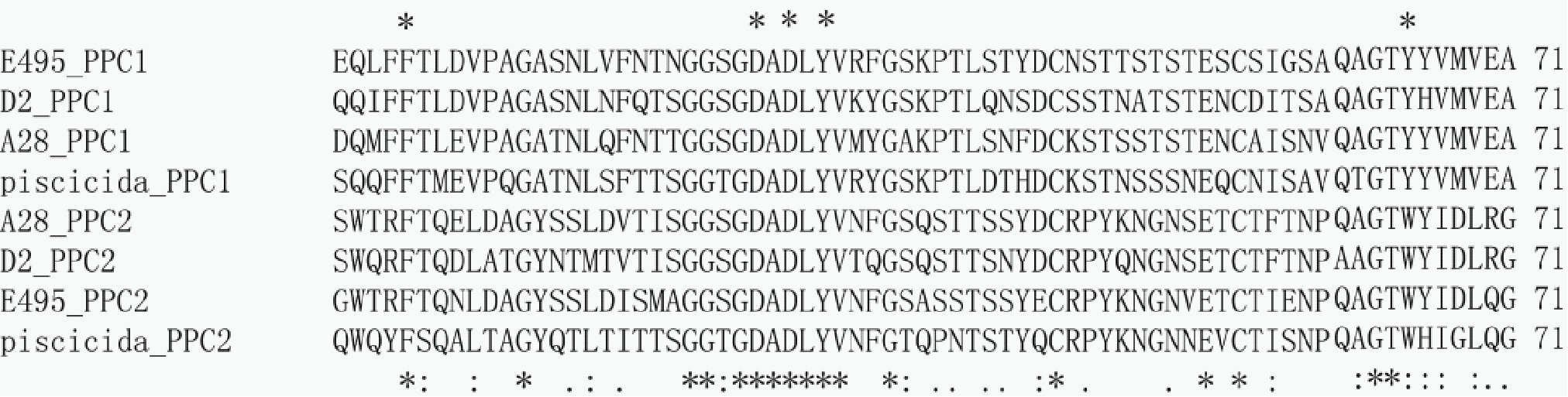

Supplement: Figure S4 — Alignment of the PPC domains of E495 with those of the metalloprotease from Ps. sp. A28, the putative metalloproteinase from Ps. tunicata D2, and the metalloprotease II from Ps. piscicida. Identical residues are indicated by asterisks. Sequences were aligned using Clustal ×1.83. (TIF) [file pone.0035442.s004.tif]

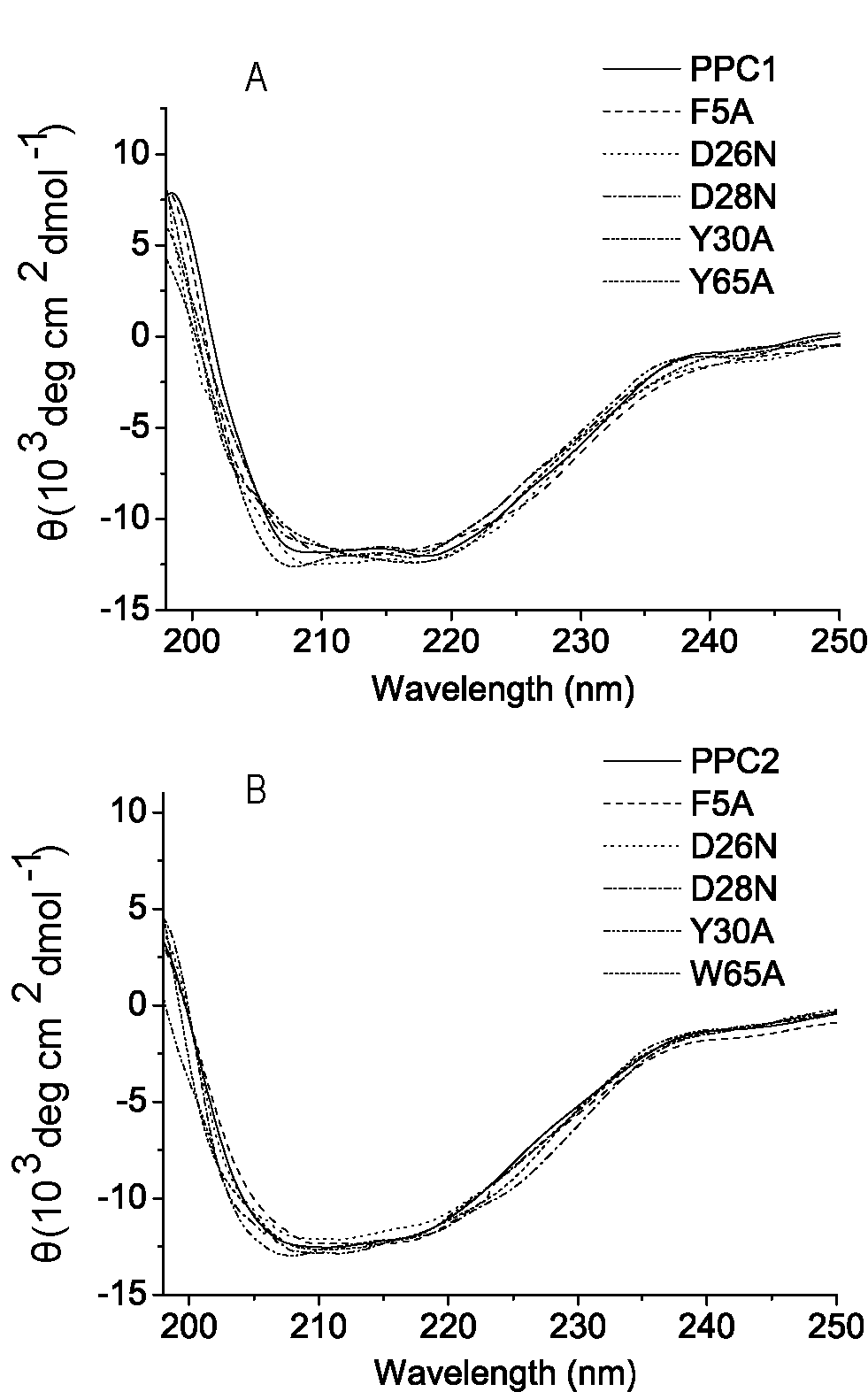

Supplement: Figure S5 — CD spectra of GST-fused PPC domains and their mutants. The spectra were measured on a Jasco J-810 spectropolarimeter (Jasco Japan) with the method described in “Materials and Methods.” (TIF) [file pone.0035442.s005.tif]
