# Supplementary material for: Structural and Functional Characterization of Mature Forms of Metalloprotease E495 from Arctic Sea-Ice Bacterium Pseudoalteromonas sp. SM495
Source: PLoS One. 2012 Apr 16;7(4):e35442. doi: 10.1371/journal.pone.0035442 (PMC3327674; doi:10.1371/journal.pone.0035442)
Supplement: Table S1 — Oligonucleotide primers used to amplify PPC domains and their mutants. (DOC) [file pone.0035442.s006.doc]

Table S1 Oligonucleotide primers used to amplify PPC domains and their mutants

| Primer | Sequence |
| --- | --- |
| PPC1 | 5’- CGGAATTCGAGCAGTTGTTTTTTAC -3’^a^ |
| PPC1_anti | 5’-GGCTCGAGTGCCTCTACCATAACG-3’ |
| PPC1F5A | 5'-CGGAATTCGAGCAGTTGTTTGCCACACT-3' |
| PPC1F5A_anti | 5’-GGCTCGAGTGCCTCTACCATAACG-3’ |
| PPC1D26N | 5'- TAATGGCGGCTCTGGTAATGCAGATTTATACGTAC-3' |
| PPC1D26N_anti | 5'-GTACGTATAAATCTGCATTACCAGAGCCGCCATTAG-3' |
| PPC1D28N | 5'-GGCGGCTCTGGTGATGCAAATTTATACGTACGATTTG-3' |
| PPC1D28N_anti | 5'-CAAATCGTACGTATAAATTTGCATCACCAGAGCCGCC-3' |
| PPC1Y30A | 5'-GGCGGCTCTGGTGATGCAGATTTAGCCGTACGATTTGGAAG-3' |
| PPC1Y30A_anti | 5'-CTTCCAAATCGTACGGCTAAATCTGCATCACCAGAGCCGCC-3' |
| PPC1Y65A | 5’-CGGAATTCGAGCAGTTGTTTTTTAC -3’ |
| PPC1Y65A_anti | 5'-GGCTCGAG TGCCTCTACCATAACGTAGGCTG-3’ |
| PPC2 | 5’-GTGAATTCGGTTGGACACGCTTTAC-3’ |
| PPC2_anti | 5’-CCTCGAGACCTTGTAAGTCTATATACC-3’ |
| PPC2F5A | 5'-GTGAATTCGGTTGGACACGCGCCAC-3' |
| PPC2F5A_anti | 5’-CCTCGAGACCTTGTAAGTCTATATACC-3’ |
| PPC2D26N | 5'-CAATGGCCGGTGGTTCTGGTAATGCTGATTTATATGTAAATT-3' |
| PPC2D26N_anti | 5'-AATTTACATATAAATCAGCATTACCAGAACCACCGGCCATTG-3' |
| PPC2D28N | 5'-GCCGGTGGTTCTGGTGATGCTAATTTATATGTAAATTTTGGTT-3' |
| PPC2D28N_anti | 5'-GAACCAAAATTTACATATAAATTAGCATCACCAGAACCACCGGC-3' |
| PPC2Y30A | 5'-CCGGTGGTTCTGGTGATGCTGATTTAGCTGTAAATTTTGGTTCAG-3' |
| PPC2Y30A_anti | 5'-CTGAACCAAAATTTACAGCTAAATCAGCATCACCAGAACCACCGG-3' |
| PPC2W65A | 5’-CGGAATTCGAGCAGTTGTTTTTTAC -3’ |
| PPC2W65A_anti | 5'-CCTCGAGACCTTGTAAGTCTATATAGGCTGTAC-3' |

^a^ EcoR I/Xho I restriction sites in the primers are underlined.
