# Supplementary material for: Structural and Functional Characterization of Mature Forms of Metalloprotease E495 from Arctic Sea-Ice Bacterium Pseudoalteromonas sp. SM495
Source: PLoS One. 2012 Apr 16;7(4):e35442. doi: 10.1371/journal.pone.0035442 (PMC3327674; doi:10.1371/journal.pone.0035442)
Supplement: Table S2 — Quantitive analysis of the amount of CPC or casein bound by the PPC domains in the assay of the “pull-down” affinity head interactiona. (DOC) [file pone.0035442.s007.doc]

Table S2 Quantitive analysis of the amount of CPC or casein bound by the PPC domains in the assay of the “pull-down” affinity head interaction ^a^.

|  | Relative amount of the unbounded protein (%) ^c^ | |
| --- | --- | --- |
|  | CPC (%) | Casein (%) |
| PPC1 | 63.6 | 71.2 |
| PPC2 | 64.8 | 76.5 |
| PPC12 | 67.9 | 76.8 |
| GST | 105 | 112 |
| Control ^b^ | 100 | 100 |

^a^ Coomassie-stained gels shown in Fig. 6 were scanned by using Epson Perfection V500 scanner (Seiko Epson, Japan) and the proteins in the gels were quantified using ImageJ 1.43u software (NIH, USA).

^b^ The amount of CPC or casein shown in lane 4 in Fig. 4 was defined as 100%.

^c^ The relative amount of CPC or casein shown in lanes 1, 2, 3 and 5 in Fig. 5 compared to the amount of CPC or casein shown in lane 4.
